# Hospital and Institutional News

**Published:** 1918-12-28

**Authors:** 


### HOSPITAL AND INSTITUTIONAL NEWS.

### THE LEAGUE OF MERCY.

The 19th annual meeting of the League of Mercy on the 20th inst. had three interesting and encouraging features, one of which was the fact, as mentioned by Lord Stamfordham in the King's letter to the meeting, that "the League had, with "the sum raised in 1918, contributed more than "£300,000 to the voluntary hospitals. The King "and Queen congratulated the President and "officers of the League on another year of steady "progress. The work done by these institutions "during the war in ministering to the relief of "wounded and sick soldiers and sailors over and "above their regular services to the civilian popula-"tion was beyond all praise, and must serve to "strengthen and encourage the support of agencies "which, like the League of Mercy, have laboured "for the maintenance of the voluntary hospitals "through the stress of war as well as in normal "times of peace. It is His Majesty's intention at an "early date to appoint the Prince of Wales to be "Grand President of the League in accordance with "article 5 of the Charter, which office was held by "the King for nine years before he ascended the "throne. The interest which their Majesties have "taken in the League of Mercy is unabated, and "they rejoice at the highly successful record "achieved since its inauguration."

### LORD FARQUHAR ON THE LEAGUE.

Viscount Farquhar, as Chairman of the meeting, declared that His Majesty's letter was an encouragement to all the workers to persevere in their efforts to promote the prosperity of the League, and all the members felt most grateful to the King for the announcement that H.R.H. the Prince of Wales was to be appointed Grand President. All present much regretted that the lady Grand President, H.R.H. Princess Alice, Countess of Athlone, was prevented by indisposition from attending to present the Order of Mercy to those whom it had been awarded. It was an additional proof of Their Majesty's gracious interest that H.R.H. Princess Mary had kindly undertaken to make these presentations on H.R.H.'s behalf. In congratulating H.H. Princess Helena Victoria on her splendid work in the four counties in which she was lady President, namely, Berkshire, Buckinghamshire, Hampshire, and Oxfordshire, this year, he regretted her absence from the meeting. H.H.'s indefatigable efforts had resulted in raising some £3,000 in these counties since the last meeting. A very successful meeting had taken place at Newmarket in the grounds of the Jockey Club, kindly lent for the occasion, under the patronage of the King. They were passing through strenuous times, requiring the output of every effort to secure that every possible penny should be collected in order to meet the urgent need of the sick civilians, and, in addition, the wounded and disabled sailors and soldiers still in our hospitals.

### SOME ZEALOUS WORKERS.

Lord Farquhar expressed genuine regret at having to announce the resignation of the Dowager Countess of Chesterfield as President of the South Kensington district where she had done such splendid work for so many years. The Dowager Countess had her heart in the work from the outset, and we can testify to the inestimable value resulting to the League from her genuine interest and inspiring example. H.H. Princess Marie Louise had consented to fill the vacancy thus caused, and will become responsible in future for the districts of North and South Kensington, of which districts she will henceforward be the President. Compliments and thanks were given to Mrs. Murdoch for the great help and continuous labour she had put into the organising work connected with the four districts of which H.H. Princess Helena Victoria was the President.

### "REFORM THE EXECUTIVE COMMITTEE."

The Chairman announced that at a meeting held at his house before the meeting, several reforms were proposed, which included regulations to secure the attendance of those who had been absent from the Executive Committee. "There should be some re-"organisation so as to get first-class people on to "the Executive Council in order to gain the con-"fidence of the public and increase the success and "prosperity of the League."

### THE SIX MOST PRODUCTIVE DISTRICTS.

The Honorary Treasurer announced that the districts which were at the head of the list of contributors during 1918 were, in order, (1) Berkshire, including profits from a concert given at Reading, £1,770; (2) Fulham, with a total of £1,750 which included a legacy from the late Mr. Philip Walker of £990; (3) South Kensington, £1,048; (4) South Paddington, £1,000; (5) Epsom and Esher, £934; and (6) Hampstead, £852, which was one of the most energetic districts owing to the work of the Rev. Hornby Steer and Mrs. Clarke, with the able co-operation of Major and Mrs. McLean. The Treasurer stated that the League had collected £21,373 this year. Other points are dealt with in an article on page 257 of the present issue.

### THE APATHY OF GLASGOW.

A kind of financial fog seems to oppress the Glasgow Hospitals. Not only do the great institutions accumulate big deficits, but it is the same with the Glasgow Royal Maternity and Women's Hospital. After three years of annual indebtedness, it now needs £15,000 to start the new year with a clean sheet. It is strange that Glasgow, one of the richest of our cities, should be so apathetic in hospital affairs, and should support its hospitals so inadequately. Glasgow must awake or forfeit its position.

### A BATH ACCIDENT.

The sudden death of Dr. George Ogilvie, the senior physician to the French Hospital, occasioned an inquest at which a verdict of accidental death was returned. It appeared from the evidence that the doctor fell while getting out of his bath, and that a fractured clavicle was followed by bronchitis. Dr. Ogilvie was sixty-six years old.

### A TRIBUTE TO A CHAPLAIN.

A gratifying proof of the regard which is sometimes felt for the work of a hospital chaplain is to be found at Cardiff, where a sum of one thousand guineas has been given to King Edward VII. Hospital out of regard for the Rev. John Morris, the late Nonconformist chaplain. The anonymous donors describe their gift as a tribute to him and to the work of an office which is sometimes thankless.

### A WORD OF PRAISE FOR ITALIAN PHARMACISTS.

The Cardinal Archbishop of Milan, on a recent visit to the wards of the Hospital Maggiore, in that city, where convalescents from the influenza were accommodated, spoke words of encouragement to the staff. In addition to their colleagues he is reported to have made special mention of the numerous pharmacists attached to the hospital institutes. These, His Eminence declared, under present circumstances, fulfilled their daily work inspired by a spirit of sacrifice and citizenship. The work and skill of the hospital pharmacists have been too little recognised in the past, and English pharmacists will welcome this public tribute to members of their profession in Italy.

### CHRISTMAS GAMES AND DECORATIONS.

It will be curious to see whether ingenuity will succeed in making any new decorations this Christmas, when materials are few. The advice to use paper is no novelty, and many decorations are stored from year to year, and depend more upon the wit of the ward sister than on their own quality for their effect. There does appear, however, to be an ingenious new game which is topical in a degree which the most ingenious might envy. We know little more of it than its name, and that is "Diplomacy," and we gather that the pieces opposed to each other do not conduct their operations in a blaze of publicity, but are as cautious as, say, a lawyer or a doctor, both of whom could not win professional respect unless they often went to work in the dark. The game does suggest a pleasant diversion for the ward, and may, as little else can, teach the players to think how far it is possible to conduct business by taking everyone into their confidence. The game, however, can hardly be so good as its title. It is said to have been invented by a diplomat!

### MR. HODGE AND HIS IDOL.

It was hardly tactful of Mr. John Hodge, the Minister of Pensions, to choose the annual meeting of the Manchester Hospital Sunday Fund for a confession of faith in the State Hospital, and the expression of a hope that one day the voluntary hospitals would be maintained by the National Exchequer. We should have thought that, after his experience of his own department and that of the others which have had their say in its affairs, Mr. Hodge, in his riper age, would have ceased to expect exaggerated benefits from State management. If the war has taught the English people anything, it should have encouraged their national dislike of being interfered with and controlled by officials; but the bureaucratic tide is always very slow to ebb. The State can do inhuman things very well, because it is itself inhuman. It can mend roads, and carry letters on a wet day, and light streets, and lay drainage. But when it comes into contact with human beings it only intensifies its inhuman character. It is nothing but a machine, and its idolisation was the real crime of Prussia. Therefore, as we take leave to think, the voluntary principle is better wherever the health or happiness or personality of the individual is concerned. The cry for efficiency is only the desire for centralisation. The voluntary institution is the only one which escapes commercialism and bureaucracy.

### MRS. IRVING ON MOTHERS' PENSIONS.

The Croydon Mothers' and Infants' Welfare Association, after listening to an address by Mrs. H. B. Irving on the subject of Mothers' Pensions, lately passed a remarkable resolution. This demanded for "every widow, deserted wife, or wife of a man totally incapacitated, who is considered a suitable guardian for her children, so long as she does her duty by them, provision by the State with all the economic necessities which are regarded as adequate for wholesome life until such time as the children individually reach the age of sixteen." Mrs. Irving declared that the above was distinct from the national question of the endowment of motherhood. During the war a pound was barely worth what ten shillings had been before the war. The woman left a widow in middle life with several young children always had a hard time. Application to the Poor-Law meant, as Mrs. Irving knew from her experience as a guardian, that the guardians might break up the home and take some of the children, and perhaps give the mother a little out-relief. The proposed Home Assistance Committee, which was to be composed of Poor Law officials, would be the relief committee over again. From the details of a case, which was not sensational but carefully recorded, Mrs. Irving argued that the cost of institutional treatment and housing for children in the care of the guardians was more, in fact, one third more, than the cost of a pension of 10s. a week for each child would have been. The curse of out-relief at present was its uncertainty. That homes should be kept together wherever possible is always desirable. For this reason the case for Mothers' Pensions is strong.

### "CAN AN INSTITUTION ADVERTISE TOO MUCH?"

A correspondent writes: "The answer to this is undoubtedly in the affirmative. After all, the benevolent public is only a section of the community. By 'benevolent' one means that part of the public which habitually gives away a portion of its income. Roughly speaking, the 'benevolent' public in the United Kingdom does not consist of more than 15 per cent. of the whole. This section is generous, but it is not foolish, and when an institution becomes reckless in its advertisements that section loses patience and the name of the institution comes 'off its list.' An advertisement in nine cases out of ten has to be paid for, and the folk who give to the funds of charitable institutions know this perfectly well. There is a part of the benevolent public that believes in advertisement and a part that disbelieves in it. When, therefore, too copious and too frequent advertising is indulged in, there is a severe penalty to pay." That is why The Hospital has often pointed out that by small well-worded and set advertisements up-to-date institutions advertise quite as usefully by "the publication of its doings."

### TWO KINDS OF ANNUAL REPORTS.

According to the compiler of the annual report of the Royal Hospital and Home for Incurables at Putney, who no doubt is Mr. Charles Cutting, the secretary, who signs it, there are two kinds of annual reports, one in which a hospital board have something to say, and the other when they have to say something. We agree that there is a great difference between the two positions, and that a whole year and nothing for the board to report would be a confession of failure, apart from the question of paper shortage. We have always been staunch upholders of the propaganda value of the annual meeting, and as the chief manifesto of the board at this meeting is the annual report, that production is worthy of the careful consideration that is evidently devoted to the one under review. There should be always something to say in respect of a progressive institution, and its supporters are always willing to congratulate themselves on their knowledgeableness in selecting a live charity for a share in what they have to give away. Further, those who have not given may be, and often are, attracted by a record of useful work prepared and presented in attractive and readable form.

### HEALTH MINISTRY AND INSTITUTIONAL OFFICERS.

Nurses and other members of the staffs at hospitals and infirmaries are wondering how the new Ministry of Health Bill will affect their status. It may be reassuring to them to know that in the proposed Bill now published the following clause is inserted: "The Minister may from time to time distribute the business of the Ministry amongst the several persons transferred or attached thereto in pursuance of this Act, in such a manner as he may think right, and those officers shall perform such duties in relation to that business as may be directed by the Minister: Provided that such persons shall be in no worse position as respects the tenure of office, salary, or superannuation allowances than they would have been if this Act had not been passed." It will thus be seen that, where present nursing staffs and other officers at infirmaries, mental hospitals, etc., are taken over (and in most cases they will be) their status and salary are to be *no less* than formerly, though of course the conditions may be better. As regards the voluntary hospitals, much will depend on the arrangements made with the Government, but it hardly seems likely that nurses will suffer loss of office through the changed administration.

### INFLUENZA AND SCHOOL CHILDREN.

The statistics are now becoming available as to the effect of the epidemic of influenza on the various ages of the patients, and it is shown that children of school age between five and fifteen years old have been best able to resist the attack. Dr. Francis Stevens, medical officer of health for Camberwell, in his monthly return, gives the number of deaths from influenza at 173. Six were under one year old, twenty-two between one and five years of age, twelve between five and fifteen years of age, twenty-six between fifteen and twenty-five years of age, ninety between twenty-five and sixty-five years of age, and seventeen over sixty-five years of age. The question was raised by Councillor G. Baty, a head-schoolmaster, as to the very small number of deaths of children of school age—between five and fifteen years old—as compared to the other periods. He characterised it as a curious fact, noticeable all over London, that children of these ages had apparently suffered less from the epidemic than any other section of the community. They had been able to resist the epidemic more successfully than those either older or younger than themselves. Dr. Stevens was unable to give any reason for this remarkable fact, but stated that the mortality from the epidemic was most severe amongst the young adult population of the country. Possibly they felt they were more vigorous than others older or younger than themselves, and did not take precautions in time, the principal being to lay up at once when the attack was first felt. "The better the people are fed," added Dr. Stevens, "the less likelihood of them being attacked, and they are better able to resist it."

### THIS WEEK'S DRUG MARKET.

This being the last drug report of the year, it is fitting to look back and see how the prices of some typical drugs have varied between the beginning of 1918 and its end. Such extraordinary advances in values as were experienced in 1916 have been of comparatively rare occurrence this year; but still there have been fluctuations within fairly wide margins, and some of these have left the final values much lower. For instance, phenacetin costs considerably less than half the price of twelve months ago; salicylic acid is 50 per cent. lower; barbitone is considerably cheaper, and so are hexamine, paraldehyde, resorcin, and salol. But, compared with pre-war prices, the values of all these are still remarkably high—for instance, phenacetin still costs about six times the pre-war price. Among the drugs which cost more than this did a year ago may be mentioned antifebrin, bromides, boric acid, citric acid, honey, menthol, and opium. We are now approaching the time when prices should steadily recede until they reach a level comparable with the pre-war figures, and this time next year we shall probably be interested in many of to-day's prices merely as one of the curiosities of the war.

